# Comparative Evaluation of Inulin and High-Ester Pectin for Microencapsulation of *Bacillus coagulans* TBC-169: Characterization and Probiotic Application in Peanut Butter Formulation

**DOI:** 10.3390/foods14132151

**Published:** 2025-06-20

**Authors:** Mengxi Xie, Yuan Tian, Liangchen Zhang, Miao Yu

**Affiliations:** Institute of Food and Processing, Liaoning Academy of Agricultural Sciences, Shenyang 110161, China; moor1112@163.com (M.X.);

**Keywords:** *Bacillus coagulans*, inulin, high-ester pectin, microcapsules, peanut butter

## Abstract

New types of functional peanut butter containing the probiotic strain *Bacillus coagulans* TBC-169 and *Bacillus coagulans* microcapsules with different wall materials were developed. After 24 h of in vitro simulated digestion, the peanut butter with high-ester pectin (group A) and inulin (group B) microcapsules still retained 5.94 ± 0.58 × 10^8^ and 1.79 ± 0.73 × 10^9^ CFU/g of *Bacillus coagulans*, respectively. Both the high-ester pectin and inulin microcapsules could be well preserved in the peanut butter substrate and stored at 4 °C and 25 °C for 120 days. The biological activities of *B. coagulans* in the two groups were 2.64 ± 0.58 × 10^10^ and 2.31 ± 0.4 × 10^11^ CFU/g, and 5.20 ± 0.10 × 10^8^ and 2.24 ± 0.11 × 10^9^ CFU/g, respectively. The addition of microcapsules improved the texture, stability, and rheological properties of the peanut butter. Differential scanning calorimetry revealed that the microcapsules showed certain binding interactions with the oil and proteins in the peanut butter. The rheological and texture tests demonstrated an improved ductility and reduced hardness and viscosity after the microcapsule addition. Targeted metabolomics identified inulin as a synergistic substrate for *Bacillus coagulans* in the probiotic peanut butter, which enhanced the functionality and stability of the microencapsulated probiotics. This study delivered essential information and parameters for the preparation of probiotic microcapsule peanut butter and laid the foundation for future research efforts geared toward the formulation, preparation, and characterization of functional peanut butter.

## 1. Introduction

Peanut butter is a product obtained through the simple processing of peanuts. During peanut butter preparation, peanuts are baked and ground, increasing the nutrient availability and enhancing the nutrient absorption and utilization in the human body [1]. However, as the standard of living improves, consumers are finding the nutritional composition and benefits of conventional peanut butter to be inadequate. In this modern era, functional foods have become extremely popular, leading to a large demand for peanut butter with probiotics and multiple functional properties.

*Bacillus coagulans* is a Gram-positive rod-shaped bacterium with the probiotic characteristics of both *Lactobacillus* and *Bacillus* [2]. *Bacillus coagulans* produces spores and can therefore resist acidic and high-temperature conditions, indicating its potential as a probiotic in food [2,3]. Nevertheless, despite some level of acid resistance [4], *B. coagulans* still undergoes a high level of activity impairment after exposure to the low pH of gastric acid [5]. Fortunately, the emergence of embedding technology has now made it possible to address this challenge. Microcapsules prepared using embedding technology can protect functional probiotics during digestion in the human body [6]. Since the wall materials of these microcapsules are generally polymers, the gastrointestinal system cannot completely digest and absorb them in a short time. In fact, the microcapsule delivery system breaks down slowly in the intestine, achieving a slow-release effect [7]. As a result, a sufficient number of probiotics can reach the target organ—the intestine—and colonize it. These probiotics can then grow in the intestine and perform key functions, such as fermentation. Therefore, the application of microcapsule technology in the research on probiotic foods has immense potential and has thus received significant attention [8].

Inulin possesses significant advantages as a wall material for probiotic encapsulation, functioning both as a dietary fiber and a prebiotic [9]. Its macromolecular polysaccharide structure composed of fructose units linked by β-(2,1)-glycosidic bonds terminating in a glucose moiety [10,11] confers resistance to hydrolysis by human and animal digestive enzymes [12]. This renders inulin an effective low-digestible carbohydrate for healthy diets. As a prebiotic, it selectively promotes the growth and metabolic activity of beneficial bacteria like *Bacillus coagulans* and increases microbial populations while lowering the colonic pH [13]. Furthermore, its excellent water solubility and gel-forming ability facilitate microencapsulation processes. Beyond its core functions in gut health, inulin demonstrates valuable functional properties in food matrices. In chocolate formulations, it improves the texture, mouthfeel, whiteness, and shelf life; sugar-free chocolate made with inulin exhibits superior viscosity, hardness, and color compared with sucrose-based versions. Inulin addition enhances the uniformity of texture and flavor in jellies while boosting the nutritional value [14]. Coating fresh-cut fruits with inulin solutions reduces browning and improves the shelf life [15], mechanical properties, and water retention. Pectin, a widely used natural anionic polysaccharide derived from low-cost sources, is extensively employed as a food additive [16]. While classified by the degree of esterification (DE) into high-methoxyl (HMP) and low-methoxyl pectin (LMP), recent research prioritized HMP for its superior functionality in acidic protein systems. Compared with LMP (which generates destabilizing electrostatic repulsions in dairy applications [17]), HMP’s lower charge density enables stronger adsorption to proteins through hydrophobic and electrostatic interactions, providing critical steric stabilization [18]. Hence, HMP can serve as a thickener, stabilizer, gelling agent, and emulsifier to alter the structural, physicochemical, and functional properties of foods [19,20].

Peanut butter is rich in minerals and vitamins and also contains abundant unsaturated fatty acids. It has long been known as “green milk” in food [21]. The alluring and unique aromatic aroma and delicate texture of peanut butter are deeply loved by consumers both at home and abroad. However, the large amount of oil present in peanut butter can lead to oil stratification due to density differences during storage. When peanut butter is placed in a stationary condition, the oil tends to separate, increasing the contact between the air and the oil and promoting fatty acid oxidation. This affects the quality of the peanut butter [22]. A food that can act as a transport substrate is often required for microcapsule transportation. Peanut butter, which is rich in oil, can serve as an effective medium to transport microcapsules. The abundance of oil in peanut butter helps with isolating oxygen, enhancing the preservation of probiotic bioactivity. Notably, it is worth exploring whether peanut butter is suitable as a transport medium for high-ester pectin and inulin microcapsules.

This study evaluated the viability of high-oleic peanut butter as a delivery vehicle for *Bacillus coagulans* microencapsulated in high-ester pectin (HMP) or inulin. We assessed the in vivo bioavailability of probiotics and monitored the bioactivity retention during storage using differential scanning calorimetry (DSC). The impact of microcapsules on the product stability was characterized through texture analysis, rheometry, laser particle sizing, and scanning electron microscopy (SEM) to determine the structural and physicochemical modifications. Furthermore, metabolomic profiling of probiotic-fortified peanut butter and microencapsulated systems identified key differential metabolites and associated metabolic pathways, which revealed colonization-driven microbial activity. These integrated analyses established the functional compatibility of microcapsules in lipid-rich matrices while providing scientific insights for engineering health-promoting probiotic peanut butter products.

## 2. Materials and Methods

### 2.1. Materials and Chemicals

High-oleic-acid-965 peanuts were purchased from the Peanut Research Institute, Liaoning Academy of Agricultural Sciences.

Bacillus coagulans TBC-169 was purchased from Henan Xingyang Biotechnology Co., Ltd. (Nanyang, China). Inulin, food grade, was purchased from the Tianjin Binhai Jebsen Chemical Co., Ltd. (Tianjin, China). High-ester pectin, food grade, was purchased from the Shanghai Maclin Biochemical Technology Co., Ltd. (Shanghai, China). Xanthan gum, food grade, was purchased from the Henan Zhongchen Biotechnology Co., Ltd. (Zhengzhou, China).

White sugar and edible salt were purchased from local supermarkets. MRS Broth medium, AGAR powder, and biochemical reagents were purchased from the Hangzhou Baisi Biological Co., Ltd. (Hangzhou, Zhejiang, China). Porcine mucous membrane protease, pseudofilament nut lipase, porcine pancreatic enzyme, and bovine bile (all analytically pure) were purchased from the Nanjing Durai Biotechnology Co., Ltd. (Nanjing, Jiangsu, China). Methanol, acetonitrile, and formic acid (chromatographic grade) were purchased from the Thermo Fisher Scientific Company (Waltham, MA, USA). Sodium hydroxide, hydrochloric acid, sodium chloride, and potassium dihydrogen phosphate (all analytically pure) were purchased from the Beijing Lambo Kangsi Biological Co., Ltd. (Beijing, China).

### 2.2. Preparation of Bacillus coagulans TBC-169 Microcapsules

We followed the method described by Raddatz et al. with slight modifications [18]. The activated probiotic suspension was centrifuged at 4 °C and 2500 r/min for 10 min, and the precipitate was collected and dispersed in normal saline. The suspension was mixed with the wall material, homogenized using a homogenizer at 12,000× *g* for 2 min, and then mixed evenly. Microcapsules that contained probiotics were then obtained via freeze-drying.

Based on the preliminary process optimization experiment, the amount of wall material added for both groups of probiotic microcapsules was 1% xanthan gum gel.

### 2.3. Preparation of Prebiotic Peanut Butter

The peanuts were roasted, ground, microencapsulated, homogenized, stirred, sterilized, and sealed to prepare the finished peanut butter product. The parameters were as follows: roasting temperature of peanuts, 160 °C; baking time, 40 min; grinding time, 30 min; microcapsule dosage, 1%; mixing time, 20 min; mixing power, 200 W.

The following groups were analyzed: KB, no added peanut butter; FB, only free *Bacillus coagulans* added; A: high-ester pectin microcapsules added; B, inulin microcapsules added.

### 2.4. Determination of the Digestive Stability of Microcapsules in Gastroenteric Fluid

According to the method described by Wei et al. with slight modifications [13], we examined the digestive stability of the microcapsules. To simulate digestion in the stomach, the pH of the sample was adjusted to 1.6 using a 1 M L-1HCL solution. Porcine mucous membrane protease and *Pseudonut* lipase were added (0.03 g per 10 mL and 0.0009 mg per 10 mL, respectively). The samples were stirred at 37 °C for 2 h (150 r/min) in an environmental incubator shaker to simulate the stomach phase. After the simulated digestion, the pH of the sample was adjusted to about 4.7 using a sterile alkaline solution (1 L containing 150 mL 1 M L-1NaOH and 14 g NaH_2_PO_4_). Bovine bile and porcine pancreatic enzyme were then added (0.1 g per 10 mL and 0.01 g per 10 mL, respectively). The samples were digested for 2 h under the above conditions (first stage of intestinal phase). After 2 h, the pH of the sample was further increased to 7.1 with the alkaline solution. In addition, the concentrations of bile and pancreatic enzymes were adjusted to 0.1 g per 10 mL and 0.1 g per 10 mL, respectively. Under these conditions, digestion was simulated for 24 h. *Bacillus coagulans* colony counts were collected using 3M plate counts at the 2, 4, 6, 9, 12, and 24 h time points. All enzymes used in the experiment were suspended in water and sterilized.

### 2.5. Storage Stability Test of Microcapsules

The prepared microcapsules were added to sterile normal saline and stored at 4 °C and 25 °C. The samples were removed at 0, 15, 30, 45, 60, 75, 90, and 120 days to obtain 3M plate counts [23].

### 2.6. Composition and Bacterial Evaluation

The protein, lipid, sodium, energy, and carbohydrate contents of the peanut butter samples were analyzed according to the protocol described by the Association of Official Analytical Chemists, 2005.

First, 25 g of peanut butter was homogenized with 225 mL of peptone water, and serial microdilutions were prepared to determine the viability of the probiotic strain and examine the microbiological safety.

*Bacillus coagulans* TBC-169 were enumerated on Coagulation Bacillus Count Medium (Haibo Biotechnology Co., Ltd., Liaocheng, Shandong, China) under aerobic incubation at 37 °C for 48 h. The colonies were counted and the results expressed as log CFU/g.

For the determination of microbiological safety, *Staphylococcus aureus* and *Salmonella* were enumerated using M17 agar colony counts (Himedia^®^, Mumbai, Maharashtra, India) following aerobic incubation at 37 °C for 48 h.

### 2.7. Examination of Structural and Rheological Properties

#### 2.7.1. SEM

Freeze-dried probiotic microcapsules were fixed on the sample table using conductive adhesive. After vertical gold-plating under a vacuum, the micro-morphology of the microcapsules was characterized using SEM (S4800, Hitachi Corporation, Chiyoda City, Tokyo, Japan.) at an acceleration voltage of 30 kV [24]. The scale ratios of magnification were 10 µm and 1 µm.

#### 2.7.2. Fourier-Transform Infrared Spectroscopy (FT-IR)

The prepared microcapsules were sifted through a 60-mesh sieve. Then, 5 mg of the sifted high-ester pectin microcapsule and inulin microcapsule gel powder was mixed with 200 mg potassium bromide powder and fully ground to ensure even mixing. The powders were pressed into transparent sheets using a tablet press, and the sheets were scanned using an infrared spectrometer (Nicolet 6700, Thermo Fisher Scientific, USA). The scanning spectral range was 4000–400 cm^−1^ and the resolution was 4 cm^−1^.

#### 2.7.3. DSC Analysis 

Crystallization and melting curves were obtained using DSC equipment (Mettler Toledo, Gieben, Zurich, Switzerland). The instrument was calibrated to the Indian standard (99.99% purity). The analysis was performed in a nitrogen atmosphere (flow rate 50 mL/min). An aluminum crucible (40 μL) was used. About 5.0 mg of the sample was used for the analysis. For the DSC, the temperature was first reduced to 0 °C and then increased to 150 °C at a rate of 10 °C/min. Subsequently, temperature curves were obtained.

#### 2.7.4. Laser Particle Size Measurement

The average particle size and particle size distribution of the emulsion were determined using the Malvern nanoscale laser particle size analyzer. The refraction angle was set at 90°, and the measurement temperature was set at 25 ± 1 °C. Measurements were obtained in the polydisperse emulsion mode. In order to avoid the influence of multiple scattering, each emulsion was diluted 100-fold with distilled water before detection.

#### 2.7.5. Analysis of Texture Characteristics

The samples were placed on the substrate of the texture analyzer (CT-3, Brookfield Corporation, Brookfield, WI, USA). The following parameters were used for evaluation: operation mode of the texture analyzer, distance measurement; target type, distance; target value, 10 mm; trigger point load, 0.07 N; measurement cycle number, 2; and test speed, 1 mm/s. The same group of samples was evaluated three times.

#### 2.7.6. Rheological Properties

The rheological properties were tested with a programmable rheometer (MCR-92, Anton Paar GMBH, Melbourne, VIC, Australia), and both steady-state and dynamic rheological analyses were performed. The initial position of the rheometer probe was 140 mm, the measurement position was 1 mm, and the sample volume was 15 mL. Before the measurement, the sample was left alone for 5 min and the excess sample at the edge of the sensor was wiped away. The temperature range was 25–40 ± 0.1 °C, and the shear rates used to compare the changes in viscosity and shear stress were 6 r/min and 60 r/min, respectively.

### 2.8. Untargeted Metabolomics Measurement

The specific operation steps were as follows: First, each 100 μL sample was mixed with 400 μL of a mixture of acetonitrile and methanol (in a ratio of 1:1) in an EP tube, which contained an isotope-labeled internal standard mixture, and continued for 30 s. Next, the mixture was subjected to ice water ultrasonic treatment for 10 min, followed by standing at −4 °C for 1 h. Subsequently, this was centrifuged at a speed of 8000 rpm for 15 min at a temperature of 4 °C. After the centrifugation, the supernatant was filtered and analyzed using ultra-high-performance liquid chromatography equipment equipped with an Acquity UPLC system (Vanquish, manufactured by Thermo Fisher Scientific) and a Q Exactive HF-X hybrid quadrupole orbital trap mass spectrometer.

Chromatographic separation was performed using an Exion LC ultra-performance liquid chromatography (UPLC) system (SCIEX) equipped with an XSelect HSS T3 column (2.5 μm, 2.1 × 150 mm; Waters) maintained at 50 °C. The mobile phase comprised (A) water that contained 0.1% formic acid and (B) acetonitrile that contained 0.1% formic acid, which was delivered at a flow rate of 0.45 mL/min. The gradient elution program was set as follows: initially held at 2% B for 0.5 min, linearly increased to 100% B over 15.0 min (0.5 to 15.5 min), maintained at 100% B for 1.5 min (15.5 to 17.0 min), rapidly returned to 2% B at 17.1 min, and held at 2% B for 3.0 min (until 20.0 min) for column re-equilibration. The autosampler temperature was maintained at 4 °C with an injection volume of 2 μL.

Mass spectrometric detection was carried out using a SCIEX triple quadrupole mass spectrometer with an electrospray ionization (ESI) source. Analyses were conducted in both positive and negative ionization modes under optimized parameters: curtain gas at 35 psi, collision gas set to medium, ion source temperature at 550 °C, ion source gas 1 (nebulizer gas) at 60 psi, and ion source gas 2 (heater/turbo gas) at 60 psi. The IonSpray voltage was set to +5500 V in positive ion mode and −4500 V in negative ion mode.

### 2.9. Data Processing and Statistical Analysis

The data from this study are presented as the average of three groups, with experimental results expressed as the mean ± standard deviation. The data analysis was performed using SPSS 16.0 software with the one-way ANOVA method, where *p* < 0.05 indicates a significant difference. The charting and fitting processing of the experimental data were completed using GraphPad Prism 8.0 and OriginPro 2018. The microbiological counts were performed on three biological replicates.

## 3. Results and Discussion

### 3.1. Nutritional Composition and Bacterial Test Results

The nutritional compositions of the four types of peanut butter are shown in Table 1. Overall, there was no significant difference in nutritional compositions between the four types of peanut butter. Hence, the addition of microcapsules had no significant effect on the nutritional composition of the peanut butter. This may have been related to the low amount of microcapsules added in this study, i.e., 1%. Notably, although the microcapsules did not improve the nutritional content of peanut butter, the overall levels of nutrients did not decrease either. This indicates that the addition of microcapsules did not cause nutrient loss.

Only *Staphylococcus aureus* was detected in the peanut butter in trace amounts. The count of *Staphylococcus aureus* in the five bottles of peanut butter in the KB group was 10 ± 7 CFU/g. This value was 8 ± 3 CFU/g in the FB group, 9 ± 6 CFU/g in group A, and 9 ± 5 CFU/g in group B. No *Salmonella* or aflatoxin AFB1 was detected in the probiotic peanut butter, indicating that it met the national safety standards.

### 3.2. Bioactivity of Bacillus coagulans Microcapsules

**Figure 1:**
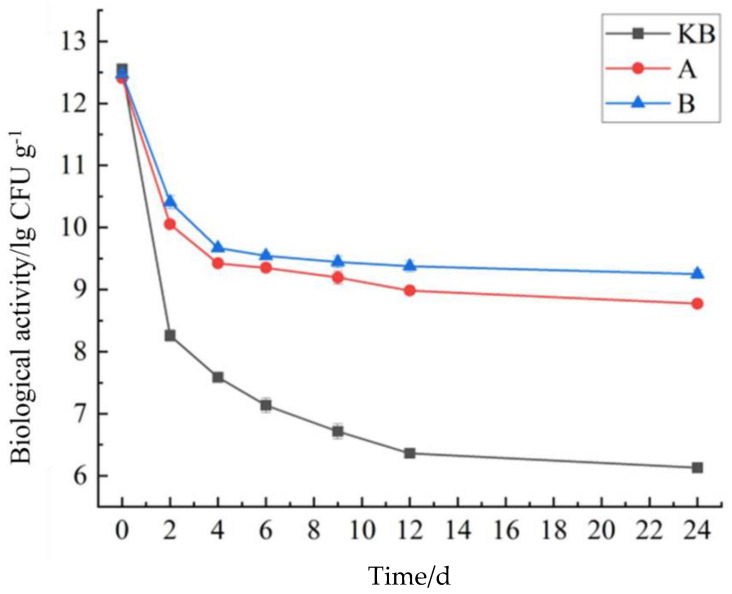
Results of in vitro peanut butter digestion.

Figure 1 shows the results of the simulated in vitro digestion of peanut butter. The bioactivity of the three groups of peanut butter carrying *Bacillus coagulans* showed a continuous decline and then tended to flatten gradually. The decline was the most rapid in the FB group, showing the same trend as the in vitro digestion of the microcapsules. During 0–2 h, due to the strong acidic conditions, the *Bacillus coagulans* activity of the peanut butter decreased very rapidly, where it dropped to 1.36 ± 0.24 × 10^6^ CFU/g—a decrease of 6 lg CFU/g. The groups A and B peanut butter samples showed good acid resistance, and their *Bacillus coagulans* biological activities at 2 h were 1.13 ± 0.07 × 10^10^ CFU/g and 1.95 ± 0.54 × 10^10^ CFU/g, respectively. Moreover, the decline in bioactivity at 6 h was less severe in groups A and B than in group FB. This was because of the inhibitory effect of cholinergic substances on *Bacillus coagulans*. The embedded microcapsules shielded *Bacillus coagulans* from the bovine bile, which prevented reductions in bioactivity. Notably, the decline trend of group B was slower than that of group A. This could have been because in group B, inulin formed a denser capsule shell due to its high viscosity and water solubility [25].

A comparison of the data between group A and group B revealed interesting findings. The initial activity of group B was not as high as that of group A. However, after 9 h of simulated in vitro digestion, group B showed higher activity, and the difference increased significantly during 12–24 h, likely due to the denser shells in group B. The *Bacillus coagulans* activity of peanut butter in the FB group was 1.36 ± 0.24 × 10^6^ CFU/g, compared with 5.83 ± 0.37 × 10^5^ CFU/g after exposure to stomach acid and choline. This two-fold difference indicated that the peanut butter had a protective effect on *Bacillus coagulans*. The *Bacillus coagulans* activities in groups A and B at 24 h were 5.88 ± 0.67 × 10^7^ CFU/g and 1.77 ± 0.73 × 10^8^ CFU/g, respectively. The 24 h *Bacillus coagulans* activities of peanut butter in groups A and B were 5.94 ± 0.58 × 10^8^ CFU/g and 1.79 ± 0.73 × 10^9^ CFU/g, respectively, which represented a 10-fold difference. This shows that peanut butter was suitable as a transport substrate for *Bacillus coagulans* microcapsules. Moreover, there was a certain mutually beneficial relationship between the peanut butter and the two types of microcapsules that could protect the activity of *Bacillus coagulans* more effectively.

### 3.3. Influence of Storage Time on the Probiotic Activity of Peanut Butter

**Figure 2:**
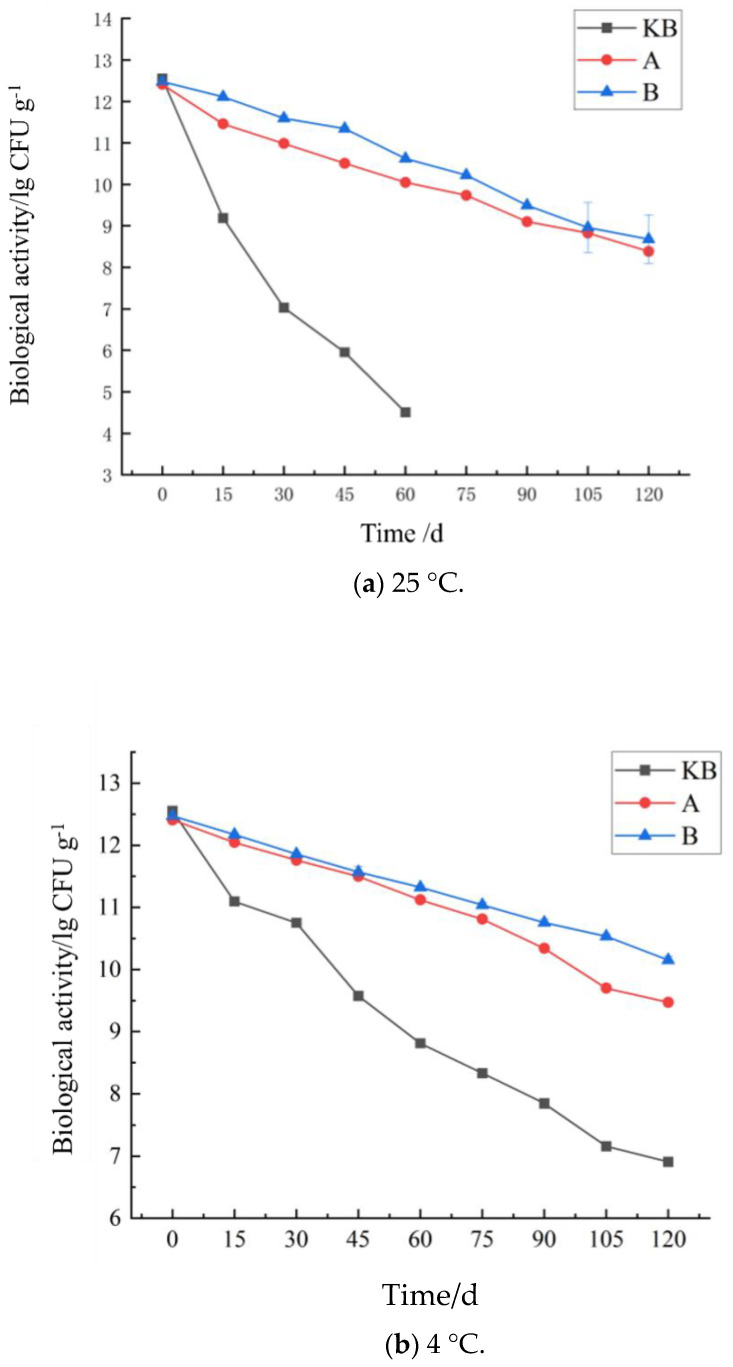
Effect of storage time on peanut butter probiotic activity at 4 °C and 25 °C.

Whether the microcapsule activity of peanut butter can be maintained to meet probiotic food standards is a key determinant of the quality of probiotic peanut butter. As we know, probiotics are adversely affected by external factors during storage [26]. Moreover, their biological activity and benefits also reduce gradually. Although inulin-embedded *Bacillus coagulans* achieved good results in our previous experiments, we noted that peanut butter could gradually decompose the wall material of the microcapsules, resulting in direct exposure to *Bacillus coagulans* and a short period of high biological activity in the probiotic peanut butter. Therefore, the storage stability of the bioactive probiotic peanut butter with microcapsules was studied again. To this end, the changes in bioactivity at 25 °C and 4 °C were examined over 120 days. As shown in Figure 2, at 25 °C, the *Bacillus coagulans* activity at 60 days was 4.6 ± 0.4 × 10^3^ CFU/g in the KB group and 3.2 ± 0.1 × 10^4^ CFU/g in the FB group. Compared with *Bacillus coagulans* directly exposed to the air, the *Bacillus coagulans* suspended in peanut butter showed an increase of 1 lg CFU/g in biological activity. However, the biological activity remained far below the internationally recognized activity standards of probiotic foods (8 lg CFU/g). By comparing the bioactivities of *Bacillus coagulans* at 25 °C between the FB group and groups A and B with probiotic microcapsules, we observed that the bioactivity of the probiotic peanut butter remained above 8 lg CFU/g after 120 days in both microcapsule groups, which is in line with internationally recognized probiotic food activity standards. Among the two microcapsule groups, group B showed better bioactivity than group A throughout the experimental period. This was primarily due to two reasons. First, the wall shell of the inulin and xanthan gum was denser. Furthermore, the viscosity of inulin was better, and the hydrogen bonds and the van der Waals forces between the inulin and xanthan were stronger than those between xanthan and high-ester pectin [25]. In addition, by comparing the bioactivity data of the microcapsules stored at 25 °C, we observed an overall activity increase of 1 lg CFU/g, which indicated that the bioactivity of peanut butter that contained microcapsules was 10-fold higher than that of free *Bacillus coagulans*. These results indicate that peanut butter was not only a good carrier substrate but also a good storage substrate for *Bacillus coagulans*. The protective effect of the microcapsules was supplemented by the protective effect of peanut butter oil on the probiotics.

The biological activity changes of peanut butter were also compared at 4 °C and 25 °C. In the FB group, after 120 days of storage at 4 °C, the biological activity still remained above 6 lg CFU/g, i.e., 100 times higher than that at 25 °C (Figure 2). This also showed that low temperature was more conducive to the storage of probiotics. Although time showed a similar overall effect on the probiotic activity of peanut butter at 4 °C, the decrease in bioactivity remained much slower at 4 °C than at 25 °C in groups FB, A, and B. In addition, at 4 °C, the gap between the biological activities of group A and group B widened gradually. That is, the value of the biological activity of group A was higher than that of group B and increased gradually, indicating that the group B microcapsules were more stable than the group A microcapsules. The FT-IR results demonstrate that the main forces of interaction between the wall materials of microcapsules A and B were hydrogen bonds and van der Waals forces, and the force in group B was stronger than that in group A. Since hydrogen bonds and van der Waals forces are both weak forces, they may weaken after long durations, resulting in the exposure of the core material and a subsequent decrease in biological activity. The storage experiment validated this point. Hence, it is possible that group A and group B microcapsules collapsed during storage. Because the interaction force of group A was weaker than that of group B, the biological activity of group B was higher than that of group A.

### 3.4. Structural and Rheological Properties

#### 3.4.1. Particle Size

**Figure 3:**
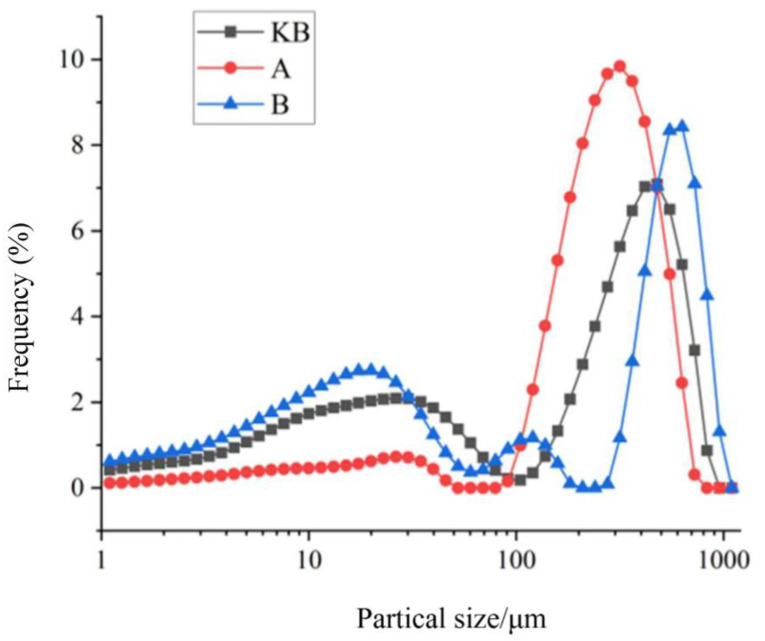
Particle size analysis of the probiotic peanut butter.

Figure 3 shows the particle size and distribution of the four groups examined using a laser particle size analyzer. The particle sizes in all three groups of peanut butter followed a normal distribution. However, the range of particle sizes was broader than that observed in the microcapsule study. This indicates that the peanut butter did not approach a uniform particle size distribution after milling, likely because specific parameters could not be controlled by the agricultural machinery. All three types of peanut butter showed two peak surface ranges: 1–100 μm and 100–1000 μm. The peak surface of the 1–100 μm range was large, indicating that the particle size distribution of the peanut butter was largely concentrated in this range. In the particle size range of 100–1000 μm, the peak plane calculation showed that the KB group occupied nearly 33% of the total distribution, while group B occupied a relatively large proportion (41%) and group A occupied a relatively small proportion (13%) of this range. This difference can be attributed partly to the addition of microcapsules. The particle size distributions of group A and group B mainly tended to remain in this range. The other primary reason for the difference was the uncontrollable inconsistency that arises during food processing with agricultural machinery.

The particle size of peanut butter is closely linked with its stability and quality. Smaller particle sizes are more likely to cause oil separation, resulting in stratification. However, larger particle sizes can impact the taste of peanut butter. Additionally, they can cause discontinuity, affect the spreadability, and reduce the ductility in peanut butter, affecting its overall quality and texture. In this study, the average particle sizes of the peanut butter were 265.338 μm (KB), 296.469 μm (A), and 303.618 μm (B). This size distribution was largely concentrated in two ranges, each with its own advantages. The small particle size could make the butter less thick, while the large particle size could enhance the adsorption function of oil, thus increasing the stability.

#### 3.4.2. DSC

**Figure 4:**
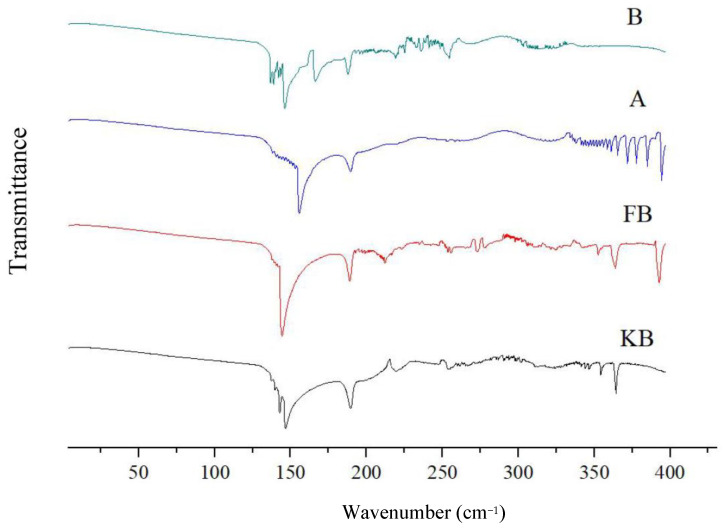
Differential scanning calorimetry of the probiotic peanut butter.

In DSC, alterations such as the disappearance of endothermic peaks, emergence of new peaks, changes in peak shapes and starting points, variations in peak temperatures/melting points, and changes in relative peak area or enthalpy occur due to interactions between the solid substances being analyzed [26]. Thus, using DSC, we examined whether the microcapsules interacted with each other after the addition to peanut butter or during storage, and whether this had a positive or negative effect on the peanut butter. The DSC results are shown in Figure 4. By comparing the changes in the peak surface among the four groups of peanut butter at 150 °C, we found that this peak surface was mainly affected by the high-temperature denaturation of the peanut protein. The peak surface was large in the KB and FB groups, indicating poor thermal stability. However, the addition of microcapsules in groups A and B obviously decreased the peak surface and increased the change temperature, demonstrating that the addition of polysaccharide microcapsules could improve the thermal stability of peanut protein. In addition, during the initial temperature change at 150 °C, both groups A and B showed a small left shift, indicating that the microcapsule polysaccharide material slightly improved the stability of the peanut butter. By comparing the peak surface changes of the peanut butter in groups A and B around 150 °C, we found that the peak surface and enthalpy changes of the peanut butter in group B were smaller, which indicates that the addition of microcapsules could enhance the stability of the peanut butter. The changes in the peak surface among the four groups around 190 °C were similar to those around 150 °C. The peak surfaces in the KB and FB groups were larger, while the surfaces in groups A and B were smaller. This may have been because some peanut protein particles were larger in these two groups and required more heat to undergo physical or chemical changes. The peak surface changes in the four groups of peanut butter around 360 °C were mainly due to the endothermic alterations in macromolecular polysaccharides, such as the molecular rearrangement caused by the endothermic absorption of dietary fiber and other substances. This change was the most obvious in group A, which was due to the larger molecular weight of high-ester pectin. Meanwhile, group B showed a relatively stable performance because the polysaccharide molecular weight was relatively low in this group.

#### 3.4.3. Texture

The addition of microcapsules had no significant effect on the elasticity and adhesion of the peanut butter (Table 2). However, it affected its hardness and viscosity. High-ester pectin- and inulin-embedded microcapsules made the peanut butter less rigid, and thus, easier to consume. The same was true for stickiness.

#### 3.4.4. Rheological Characteristics

**Figure 5:**
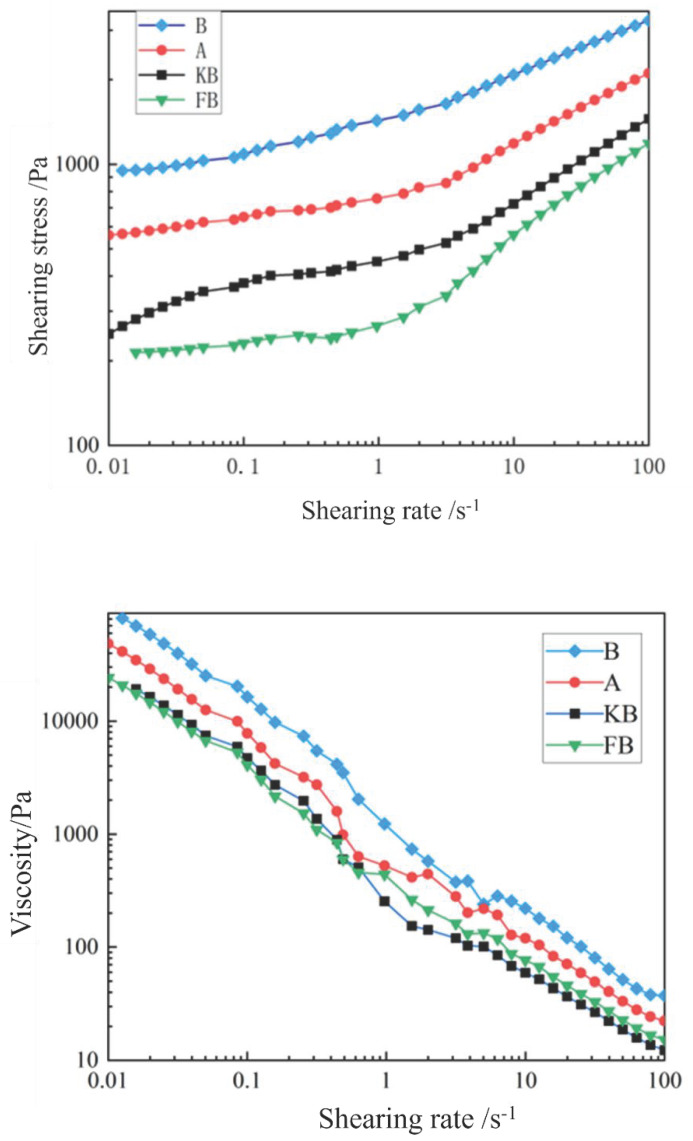
Rheology diagrams of the probiotic peanut butter.

Origin software was used to smoothen the shear stress and viscosity data (Figure 5). The relationship between the shear rate and shear stress reflects the flow characteristics of a sample. In a pseudoplastic fluid like peanut butter, with the increase in the shear rate, the shear stress gradually reduces. This behavior can be attributed to the relatively tight structure of proteins, fats, and sugars in peanut butter [27]. In the presence of an external shear force, the spatial network structure of peanut butter is partially disrupted, which weakens the interaction between molecules and reduces its viscosity [28].

In this study, there were no significant differences in the shear stresses and viscosities between the KB and FB groups, indicating that the addition of *Bacillus coagulans* alone had little effect on the rheological characteristics of the peanut butter. This may have been because the small particles of *Bacillus coagulans* and peanut butter could not be combined during the shear process. The shear stresses and viscosities of group A and group B were significantly better than those of group KB. Hence, the addition of the microcapsules optimized the shear stresses and viscosities of the peanut butter in both groups. Notably, the viscosity and shear stress showed increases in group A because high-ester pectin has a long molecular chain. These pectin molecules crosslink and interact with mineral ions (such as calcium) and other substances in peanut butter to form a more stable network structure, thus increasing the viscosity and shear stress of peanut butter [29]. Group B had a higher shear stress and viscosity than group KB, possibly due to the formation of more hydrogen bonds, which promoted the formation of a more stable network structure [20]. Notably, the shear stress and viscosity values were higher in group B than in group A, likely because hydrogen bonds are stronger than the interactions and crosslinking bonds between long molecular chains and mineral ions.

### 3.5. Metabolomic Differences Between High-Ester Pectin and Inulin Microencapsulation

**Figure 6:**
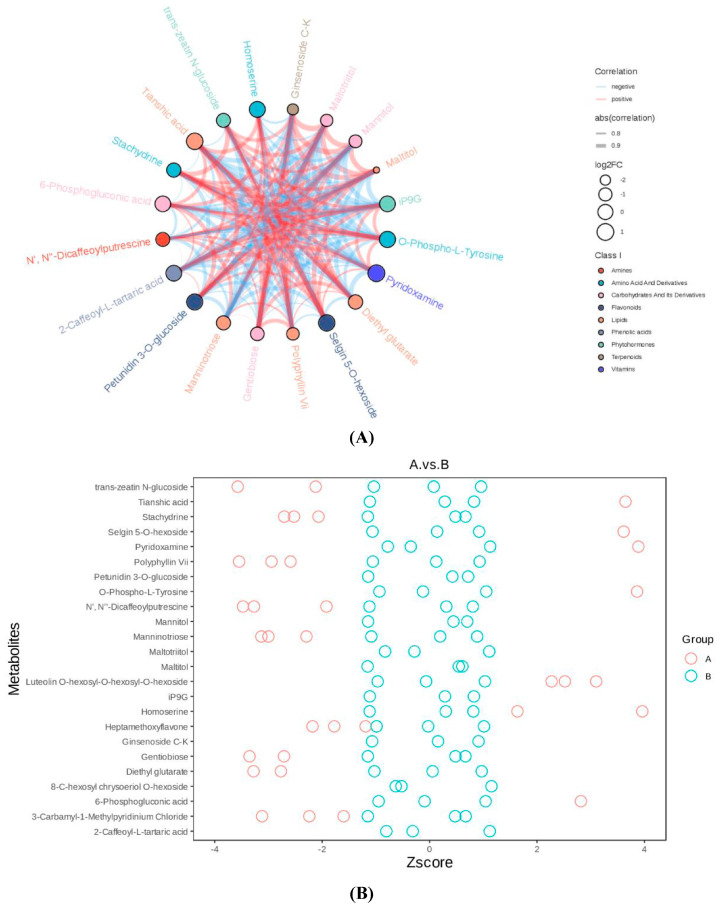

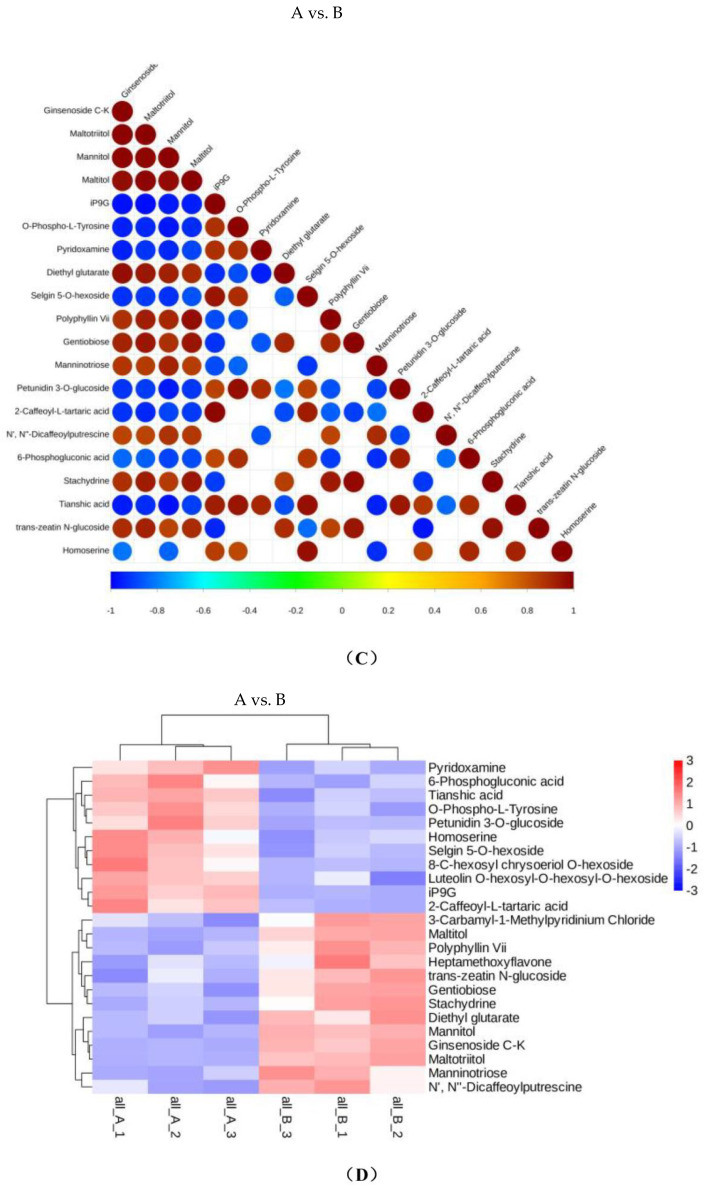

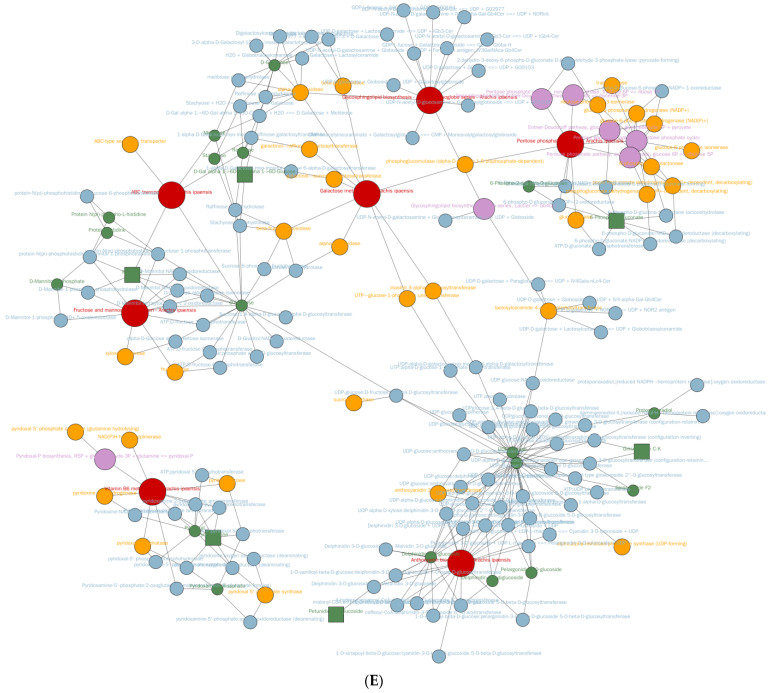
Differential metabolites between groups A and B. (**A**) Chord diagram of metabolite interactions; (**B**) Z-score distribution of differential metabolites; (**C**) correlation matrix of metabolites; (**D**) intergroup clustering heatmap; (**E**) KEGG pathway regulatory network.

LC-MS-based pseudo-targeted metabolomics data were validated through stringent quality control (QC), with QC samples demonstrating high instrument stability (total ion chromatogram overlap, Figure 6A) and experimental reproducibility. A total of 743 differential metabolites were identified, predominantly amino acids (22.8%) and carbohydrates (12.0%) (Figure 6B). High-methoxyl pectin microencapsulation (group A) upregulated 124 metabolites, including neuroactive acetylcholine and anti-inflammatory luteolin derivatives. Inulin-microencapsulation (group B) induced the most pronounced changes (167 metabolites, 156 upregulated), which enriched anti-inflammatory mannitol, CYP450-inhibiting chrysoeriol glycosides, and gut-modulatory spermidine (Figure 6C). The correlation analysis highlighted a robust synergy in inulin-microencapsulated systems (group B), where mannitol and spermidine showed near-perfect correlations (r = 0.8–1.0), whereas pectin (group A) exhibited weaker interactions (Figure 6D). KEGG enrichment identified amino acid metabolism (phenylalanine/tyrosine) as the dominant pathway (*p* < 0.05), which was driven by the *B. coagulans*-mediated proteolysis of peanut proteins into bioactive amines. Integrated network modeling revealed inulin’s heightened prebiotic capacity, which demonstrated broader metabolic reprogramming effects across 19 pathways (64 pathway-associated metabolites) relative to pectin’s 17-pathway modulation (41 metabolites) (Figure 6E).

The metabolic divergence between microencapsulated and free *B. coagulans* underscores the critical role of wall materials in modulating probiotic functionality. Inulin’s superiority over high-methoxyl pectin aligns with its fermentable fiber properties, which enhance bacterial survival and metabolic activity in the gastrointestinal tract. The upregulation of lactobionic acid (antioxidant) and spermidine (anti-aging) in group B suggests potential health benefits, including improved lipid metabolism and gut barrier function [30]. Conversely, the reduction in aroma-related metabolites in FB and group A highlights a trade-off between probiotic efficacy and sensory quality. The prominence of amino acid metabolism pathways reflects *B. coagulans*’ proteolytic activity, converting peanut proteins into bioactive small molecules (e.g., 2-phenylethylamine, indole) with putative roles in neuroregulation and anti-inflammation. Inulin’s ability to activate broader metabolic networks (19 pathways vs. 17 in pectin) further positions it as a prebiotic matrix for optimizing functional foods. These findings not only validate inulin-microencapsulated peanut butter as a promising vehicle for probiotic delivery but also provide mechanistic insights into microbial–metabolic interactions in lipid-rich matrices, advancing strategies for designing foods targeting metabolic health.

## 4. Conclusions

In this work, high-ester pectin and inulin microcapsules were prepared. The process involved the pre-homogenizing emulsification and freeze-drying embedding of *Bacillus coagulans*. Subsequently, the two types of microcapsules were added to peanut butter to prepare probiotic peanut butter. The particle size, morphology, and stability of peanut butter and probiotic peanut butter were studied. The results show that probiotic peanut butter had better digestibility in vitro. Moreover, peanut butter was very suitable as a substrate for carrying microcapsules, increasing the biological activity of *Bacillus coagulans* by 10-fold under simulated intestinal conditions and exhibiting good morphological characteristics and long-term stability. The addition of microcapsules could improve the texture, stability, and rheological properties of peanut butter. Moreover, DSC showed that the microcapsules exhibited a certain binding effect with the oil and protein in peanut butter. Untargeted metabolomics analysis demonstrated that the inulin acted as a synergistic substrate for *Bacillus coagulans* in probiotic peanut butter, where it enriched anti-inflammatory and gut-modulatory metabolites while enhancing amino acid metabolism pathways. This study provides a reference for the development of probiotic microcapsules and could guide new strategies for the production of functional peanut butters.

## Figures and Tables

**Table 1:** Nutritional information of the probiotic peanut butter.

Group	Energy J/100 g	Protein g/100 g	Lipid g/100 g	Carbohydrates g/100 g	Sodium mg/100 g
KB	2527 ± 125	21.5 ± 3.5	46.6 ± 7.2	25.7 ± 3.1	293 ± 21
FB	2592 ± 137	21.6 ± 2.7	49.6 ± 6.9	22.9 ± 2.7	287 ± 27
A	2584 ± 164	21.5 ± 3.2	50 ± 5.9	21.7 ± 2.8	302 ± 32
B	2583 ± 143	21.5 ± 2.9	49.5 ± 6.2	22.7 ± 2.5	291 ± 43

**Table 2:** Texture characteristics of the probiotic peanut butter.

Group	Elastic Index	Hardness/g	Gumminess/g	Viscosity/mJ
KB	0.97 ± 0.01 a	44.5667 ± 2.54 a	30.5333 ± 4.02 a	2.7100 ± 0.12 a
FB	0.97 ± 0.03 a	40.5667 ± 0.57 b	30.9 ± 1.47 a	2.3867 ± 0.21 ab
A	0.97 ± 0.03 a	38.3333 ±0.65 bc	36.3333 ± 1.42 a	2.1500 ± 0.04 b
B	0.9767 ± 0.03 a	36.3333 ± 0.81 c	27.3667 ± 2.99 a	2.1667 ± 0.17 b

All values shown are mean ± standard deviation. Data with the same letter in an index are not significantly different (*p* < 0.05).

## Data Availability

The original contributions presented in this study are included in the article. Further inquiries can be directed to the corresponding author.
